# Synthesis of Novel Cavitand Host Molecules via Palladium-Catalyzed Aryloxy- and Azidocarbonylation

**DOI:** 10.3390/molecules27238404

**Published:** 2022-12-01

**Authors:** Akash, László Kollár, Tímea R. Kégl

**Affiliations:** 1 Department of General and Inorganic Chemistry, University of Pécs, H-7624 Pécs, Hungary; 2 ELKH-PTE Research Group for Selective Chemical Syntheses, H-7624 Pécs, Hungary; 3 János Szentágothai Research Centre, University of Pécs, H-7624 Pécs, Hungary; 4 National Laboratory of Renewable Energy, University of Pécs, H-7624 Pécs, Hungary

**Keywords:** aryloxycarbonylation, azidocarbonylation, homogeneous catalysis, cavitand

## Abstract

Novel, elongated, resorcine[4]arene-based cavitands were synthesized via various consecutive reaction steps, including homogeneous catalytic aryloxy- and azidocarbonylation processes. The effects of carbon monoxide pressure and temperature on the conversion were examined in aryloxycarbonylation. It was revealed that a reaction temperature of 100 °C is required to achieve complete conversion both with monodentate (PPh_3_) and bidentate (Xantphos) phosphines at different carbon monoxide pressures (1–40 bar). Using ten different phenols as *O*-nucleophiles, partial hydrolysis of the esters to the corresponding carboxylic acids took place—i.e., 58–90% chemoselectivities toward esters were obtained. Moreover, the influences of temperature, reaction time and the catalyst ratio on the selectivity and conversion were described in the case of azidocarbonylation reaction. The formation of the acyl azide with high chemoselevtivity can be achieved at room temperature only. The higher reaction temperatures (50 °C) and higher catalyst loadings favor the formation of the primary amide. The characterization of the target compounds (esters and acyl azides) was carried out by IR and ^1^H and ^1^3C NMR. The discussion of the influences of various parameters is based on in situ NMR investigations.

## 1. Introduction

Since the 1980s, supramolecular chemistry has become a dynamically developing field. Recently, many host molecules have become known, of which cavitands are the most promising in many fields of application, such as gas sensors, nanoreactors and drug delivery systems [1,2,3,4]. Cavitands [5] are bowl-shaped or tubular macromolecules, which possess a well-formed large hydrophobic cavity. The formation of macromolecules based on the molecular self-assembly is well-known from biology; in this way, numerous host molecules can be prepared. During the synthesis of cavitands, predominantly traditional organic chemical reactions are applied; there are hardly any examples of homogeneous catalytic processes in the literature [6,7,8,9,10,11,12].

In the last decade, our research group had developed and described several palladium- and copper-catalyzed homogeneous catalytic processes on a cavitand scaffold, including the palladium-catalyzed aminocarbonylation [13], copper-catalyzed azide-alkyne cycloaddition (CuAAC) [14,15] and Sonogashira or Suzuki-Miyaura coupling [16]. It has been proven that homogeneous catalytic processes can be easily adopted for cavitands, and similar results can be expected in terms of both reactivity and selectivity as in the case of small molecules.

In addition to the widely used alkoxycarbonylation of aryl halides and iodoalkenes [17,18,19,20,21], and their synthetic surrogates (aryl triflates and enol triflates), aryloxycarbonylation has become a well-established and long-applied homogeneous catalytic reaction [22,23,24,25,26,27,28]. While the application of alcohols and phenols as *O*-nucleophiles in transition metal-catalyzed carbonylations is widely known and can be considered as a general synthetic tool, azidocarbonylation is still a recent discovery, dating back only a few years [29,30]. Grushin’s seminal work on palladium-catalyzed azidocarbonylation of iodoaromatic compounds was published in 2012; that is why it is not considered strange (unlike aryloxycarbonylation) that on macromolecules, to the best of our knowledge, it has not yet been applied. Some sporadic results were published on the use of azide as a *N*-nucleophile in palladium-catalyzed homogeneous catalytic reactions [31,32,33,34], revealing the high reactivity of acyl azides. Primary carboxamides as stable final products were obtained using this protocol. Continuing our investigations on the synthesis of functionalized cavitands [13,15,16,35], with the aim of their application in host-guest chemistry, homogeneous catalytic reactions were applied for the introduction of novel functionalities. The synthetic strategy of these supramolecular entities is based on the combination of high-yielding conventional procedures and highly selective homogeneous catalytic reactions.

In this study, we performed palladium-catalyzed aryloxy- and azidocarbonylation processes carried out on an cavitand scaffold, which resulted in the formation of valuable ester, amide and acyl azide functionalities on the upper rim (’inlet’) of 2-methylresorcinarene-based macrocycles of supramolecular interest.

## 2. Results and Discussion

### 2.1. Synthesis of Starting Material

**Figure 1:**
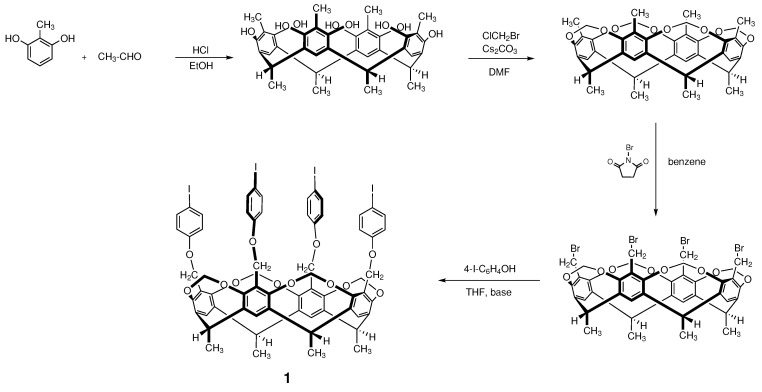
Synthesis of starting material (cavitand **1**).

Cavitand **1**, bearing four iodoaromatic functional groups on the upper rim, is highly applicable as a starting material of homogeneous catalytic processes. The synthesis of this compound is a four-step consecutive reaction sequence (Figure 1) started from small organic molecules. The first step is an acid-catalyzed condensation reaction of 2-methylresorcinol and acetaldehyde [36], the second step is alkylating the hydroxyl groups with bromochloromethane [37], the third step is a radical bromination of the methyl groups on the upper rim [38] with *N*-bromosuccinimide and the last step is a Williamson etherification with 4-iodophenol [39]. Each step can be performed with excellent (70–80%) yield.

### 2.2. Aryloxycarbonylation on Cavitand Scaffold

#### 2.2.1. Effect of Temperature and Pressure on the Conversion

In order to determine the optimal reaction conditions, phenoxycarbonylation experiments were performed with the starting material (cavitand **1**) and phenol at different temperatures (50, 70, 100 °C) and pressures (1, 10, 40 bar). Since palladium-based catalysts exhibit the highest efficiency for aryloxycarbonylation [22,23,24,25,26], palladium(II) acetate was chosen as a catalyst precursor with two different phosphines (triphenylphosphine and Xantphos). Results of temperature-dependent experiment can be seen in Table 1. At 50 °C, the conversion was negligible, and at 75 °C, it was quite low also. On the other hand, at 100 °C, the conversion was full under 24 h for both phosphine.

In contrast to temperature, the conversions achieved in 24 h at different pressures showed no differences. That is, practically complete conversions were observed with both phosphines under 1, 10 and 40 bar carbon monoxide pressures. Although differences in shorter reaction times might be expected, due to synthetic reasons, no further experiments were carried out with the excellent yields in our hands.

#### 2.2.2. Aryloxycarbonylation with Ten Different Phenols

**Figure 2:**
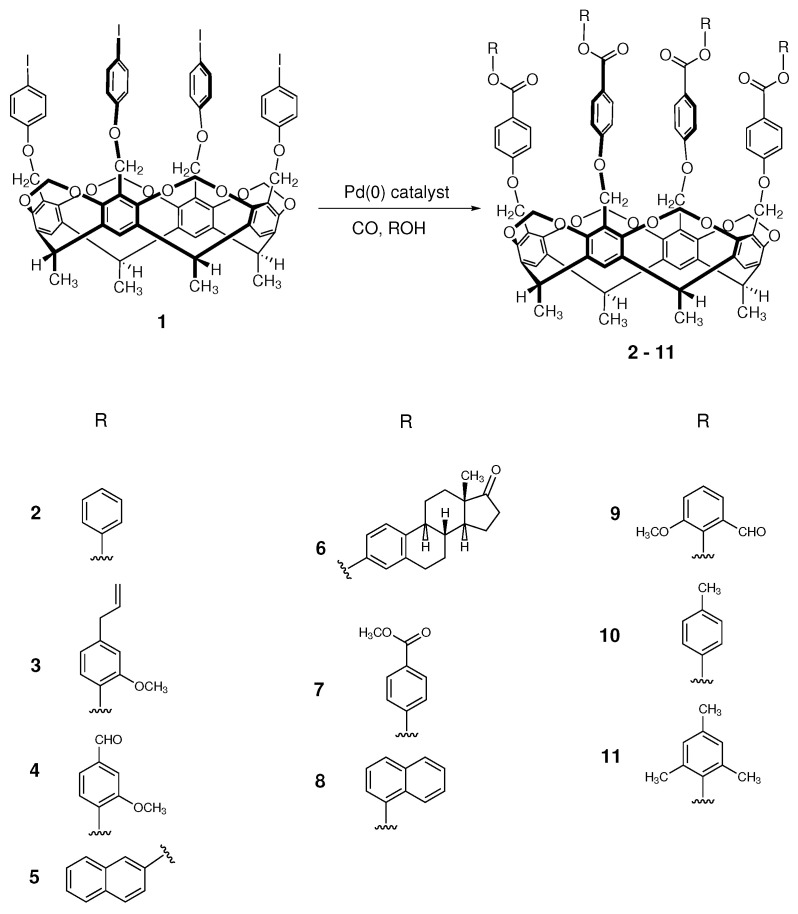
Scheme of aryloxycarbonylation reaction on the cavitand scaffold.

In light of the preliminary experiments above, 100 °C, atmospheric pressure and 24 h reaction time were applied for all the aryloxycarbonylation reactions in the presence of triethylamine as base and DMF as solvent. Moreover, ten different phenols were investigated as nucleophiles (Figure 2): phenol (cavitand **2**), eugenol (cavitand **3**), vanillin (cavitand **4**), 2-naphtol (cavitand **5**), estrone (cavitand **6**), methyl 4-hydroxybenzoate (cavitand **7**), 1-naphtol (cavitand **8**), *o*-vanillin (cavitand **9**), *p*-cresol (cavitand **10**) and 2,4,6-trimethylphenol (cavitand **11**).

The substrate was fully converted in all cases, but besides the targeted esters, side-products were formed in small amounts due to the ester hydrolysis (carboxylic acid formation). That is why the chemoselectivity was 100% in only one case (cavitand **7**, Xantphos ligand); in the other cases, a lower chemoselectivity value (58–90%) was achieved (Table 2). In general, the bidentate ligand (Xantphos) produced better chemoselectivity values, except for two cases (cavitand **4** and **10**) where triphenylphosphine proved to be more efficient.

Deepened cavitands have a strong inclination to bind to large-surface materials (due to their size and functionalized upper rims), which causes a spectacular loss in yield. Therefore, it is difficult to purify them by column chromatography, or even to use a desiccant after aqueous extraction. Hence, chemoseletivity values were determined based on in situ NMR investigations.

### 2.3. Azidocarbonylation on Cavitand Scaffold

In the papers published by Grushin and his colleagues in 2012 and 2014 [29,30], the azidocarbonylation reaction was comprehensively studied, including the mechanism also. In their work, several phosphines (as ligands) and substituted iodoaromatic compounds (as a substrate) were tested; moreover, the catalyst deactivation (catalyst poisoning) and the difficulty of achieving 100% conversion were detailed.

**Figure 3:**
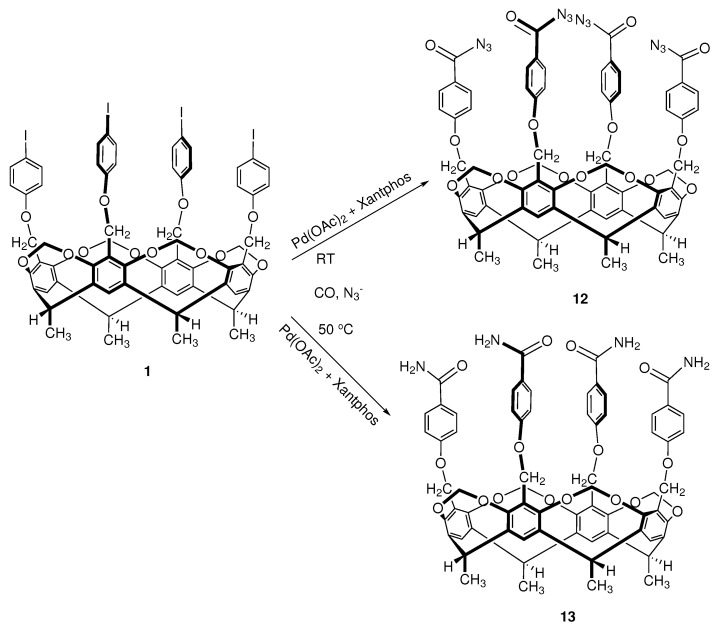
Scheme of azidocarbonylation reaction on a cavitand scaffold.

Based on Grushin’s experiments, the reaction conditions of azidocarbonylation reaction performed on cavitand **1** were carefully considered. Palladium(0) catalyst, formed in situ from palladium(II) acetate and Xantphos, was applied as catalyst, and the reaction was performed at two temperatures (room temperature and 50 °C), with different reaction times and catalyst/substrate ratios. The reaction scheme is shown in Figure 3.

In the reaction carried out at 50 °C, the amide product (cavitand **13**) was reproducibly obtained with 100% conversion and chemoselectivity (Table 3). The carbamoyl groups introduced on the upper rim are capable of forming hydrogen bonds, and different guest molecules can be selectively complexed. In this way, the cavitand can act as a molecular receptor. Furthermore, the sterically unhindered carbamoyl groups on the upper rim also can develop the opportunity for the formation of intermolecular (dimer) capsules (between two cavitand units), which have practical importance in supramolecular chemistry [40,41,42].

The production of the aroyl azide derivative (cavitand **12**) was only achieved in the room temperature reaction. The reaction time was 16 h to 3 days, the catalyst ratio was 2–8 mol%, and the conversion was 32–66% complete. In order to improve the conversion, the catalyst ratio was increased to 4 mol%, then to 8 mol%, and zinc powder was added as suggested by Grushin to reactivate the catalyst complex. Furthermore, trying to achieve full conversion, a longer reaction time (2 or 3 days) was applied also in different experiments. Unfortunately, though complete conversion was achieved, chemoselectivity values were moderate (Table 3). With a 4 mol% catalyst ratio and 16 h reaction time, the aroyl azide product (cavitand **12**) was formed with 100% chemoselectivity, and the conversion rate was 51%. By increasing the catalyst ratio to 8 mol%, 66% conversion was achieved, but the further increase in the catalyst ratio and the long reaction time resulted in the appearance of an amido-cavitand (cavitand **13**) even in the room temperature reaction. The formation of cavitand **13** can be explained by the Curtius rearrangement of the acylazide to isocyanate, followed by the reaction with traces of water. It is worth noting that primary amides with various structures could be isolated as main products from an azidocarbonylation reaction when aqueous biphasic solvent mixtures were used [33]. The other solution proposed by Grushin et al. to achieve 100% conversion, the use of a two-phase solvent system, was not applicable, since the cavitand substrate does not dissolve in apolar solvents. Moreover, hexane is routinely used to precipitate a cavitand during the cleaning process. Halogenated organic solvents cannot be used for Pd(0) complexes, because they react with the active catalyst by oxidative addition.

## 3. Conclusions

2-Methylresorcine-based cavitands with aryloxycarbonyl (aryl ester) functionalities on the upper rim were synthesized from the corresponding tetraiodo compounds in moderate to excellent yields. It was proved that a 100 °C reaction temperature is required to achieve complete conversion for both phosphines at different carbon monoxide pressures. Using ten different phenols as *O*-nucleophiles, 58–90% chemoselectivities toward esters were obtained. Palladium(0) Xantphos catalytic systems, formed in situ from a palladium(II) acetate precursor, proved to be highly active not only in aryloxycarbonylation but in azidocarbonylation as well. The latter reaction provided acyl azide at lower temperatures and supramolecular primary amide derivative at higher temperatures. Moreover, the influences of temperature, reaction time and the catalyst ratio on the selectivity and conversion were described in the case of the azidocarbonylation reaction. The formation of the acyl azide with high chemoselevtivity can be achieved at room temperature only. Higher reaction temperatures (50 °C) and higher catalyst loadings favor the formation of the primary amide.

## 4. Experimental

### 4.1. General Information

Chemicals were purchased from Sigma-Aldrich Kft, Budapest, Hungary. ^1^H- and ^1^3C-NMR spectra were recorded at 25 °C in DMSO-d_6_ on a 500 MHz Bruker spectrometer. The ^1^H chemical shifts (δ), reported in parts per million (ppm) downfield to TMS, are referenced to the residual protons (2.50 for DMSO-d_6_). The ^1^3C chemical shifts are referenced to the carbon resonance of DMSO-d_6_ (39.52 ppm). MALDI-TOF spectra were obtained on an Autoflex II TOF/TOF spectrometer (Bruker Daltonics, Bremen, Germany) in positive ion mode, using a 337 nm pulsed nitrogen laser (accelerating voltage: 20.0 kV, matrix: DHB). The IR spectra were taken in KBr pellets using an IMPACT 400 spectrometer (Nicolet) with a DTGS detector in the region of 500–4000 cm^−1^; the resolution was 4 cm^−1^. The amount of the samples was ca. 0.5 mg.

### 4.2. Synthesis and Characterization of Cavitand ***2***–***13***

#### Aryloxycarbonylation

Cavitand **1** (100 mg, 0.066 mmol), palladium acetate (2.5 mg, 0.01 mmol), triphenylphosphine (5.24 mg, 0.02 mmol) or Xantphos (6 mg, 0.1 mmol) and the proper phenol (0.3 mmol) were weighed into a three-necked round-bottom flask equipped with a condenser, a magnetic stirrer, a ball filled with argon gas and a vacuum/gas inlet. The solid components were dissolved in dimethylformamide (10 mL) under argon counterflow; then triethylamine base (110 μL, 0.8 mmol) was added to the reaction mixture. Then, the argon atmosphere was changed to carbon monoxide and the reaction mixture was stirred at 100 °C for 24 h.

The reaction mixture was filtered on filter paper, and the solvent was removed with vacuum evaporation. The residue was dissolved in CH_2_Cl_2_ treated with methanol, the resulting precipitate was collected by filtration and dried in vacuo.

#### Azidocarbonylation

Cavitand **1** (200 mg, 0.066 mmol), palladium acetate (2–8 mol%), Xantphos (2–8 mol%) and sodium azide (20.6 mg, 0.32 mmol) were weighed into a three-necked round-bottom flask equipped with a condenser, a magnetic stirrer, a ball filled with argon gas and a vacuum/gas inlet. The solid components were dissolved in THF/water (30 mL/1 mL) mixture under argon counterflow. Then the argon atmosphere was changed to carbon monoxide and the reaction mixture was stirred at room temperature (cavitand **12**) or 50 °C (cavitand **13**) for 48 h.

The reaction mixture was filtered on filter paper, and the solvent was removed with vacuum evaporation without heating. The residue was dissolved in CH_2_Cl_2_ and was treated with *n*-hexane. The resulting precipitate was collected by filtration and dried in vacuo without heating.

**2**: Dark brown powder (49 mg, 54%), mp 281 °C. IR [cm^−1^] $\nu_{max}$(KBr): 974, 1008, 1094, 1168, 1198, 1251, 1298, 1476, 1493, 1509, 1606, 1734, 2970 cm^−1^; ^1^H-NMR (500.15 MHz, CDCl_3_): 1.88 (12H, d, J 6.2 Hz, C*H*3CH ), 4.7 (4H, d, J 8.1 Hz, inner OC*H*2O), 5.06 (8H, s, ArC*H*2O), 5.14 (4H, q, J 6.3 Hz, CH_3_C*H*), 5.82 (4H, d, J 8.1 Hz, outer OCH2O), 7.03 (8H, d, J 6.0 Hz, Ar), 7.05–7.47 (24H, m, Ar), 8.19 (8H, d, J 8.3 Hz, Ar). ^1^3C-NMR (125.78 MHz, CDCl_3_): 16.2 (*C*H_3_CH), 31.3 (CH_3_*C*H), 46.2, 60.9 (O*C*H_2_O), 100.3 (Ar*C*H_2_O), 114.8, 120.9, 121.7, 125.7, 128.3, 129.4, 132.4, 139.1, 151.0, 154.0, 162,8, 164.6 (Ar*C*=O).
**3**: Light brown powder (34 mg, 32%), mp 287 °C. IR [cm^−1^] $\nu_{max}$(KBr): 974, 1008, 1068, 1250, 1475, 1511, 1605, 1734, 2971 cm^−1^; ^1^H-NMR (500.15 MHz, CDCl_3_): 1.87 (12H, br s, C*H*3CH), 3.41 (8H, br s, ArC*H*2), 3.76 (12H, br s, ArOC*H*3), 4.68 (4 H, d, J 6.6 Hz, inner OCH2O), 5.03 (8H, s, ArCH2O), 5.12 (4H, br s, CH_3_C*H*), 5.82 (4H, m, C=CH), 5.99 (4H, d J 6.9 Hz, outer OC*H*2O), 6.00 (4H, m, C=C*H*), 6.81 (4H, m, CH_2_CH=C*H*2), 7.03–8.2 (20H, m, Ar). ^1^3C-NMR (125.78 MHz, CDCl_3_): 16.2 (*C*H_3_CH), 31.3 (CH_3_*C*H), 40.1, 55.9, 60.6 (O*C*H_2_O), 100.1 (Ar*C*H_2_O), 112.8, 114.2, 116.0, 120.9, 122.1, 122.6, 122.8, 132.5, 137.2, 138.3, 139.1, 151.2, 154.1, 162.7, 164.3 (Ar*C*=O).
**4**: Beige powder (54 mg, 50%), mp 240 °C. IR [cm^−1^] $\nu_{max}$(KBr): 974, 1008, 1068, 1250, 1475, 1510, 1604, 1701, 1735, 2942 cm^−1^; ^1^H-NMR (500.15 MHz, CDCl_3_): 1.87 (12H, br s, C*H*3CH), 3.85 (12H, s, AROC*H*3), 4.68 (4H, br s, inner OC*H*2O), 5.05 (8H, s, ArC*H*2), 5.12 (4H, br s, CH_3_C*H*), 5.81 (4H, br s, outer OC*H*2O), 7.03–8.17 (28H, m, Ar), 9.98 (4H, s, ArC*H*O). ^1^3C-NMR (125.78 MHz, CDCl_3_): 16.2 (*C*H_3_CH), 31.2 (CH_3_*C*H), 56.5, 60.9 (O*C*H_2_O, 100.0 (Ar*C*H_2_O), 110.9, 114.2, 122.0, 124.6, 128.3, 129.2, 132.7, 139.1, 145.2, 12.3, 159.2, 165.6 (Ar*C*=O), 190.9 (*C*HO).
**5**: Light brown powder (60.2 mg, 54%), mp 225 °C. IR [cm^−1^] $\nu_{max}$(KBr): 976, 1008, 1057, 1240, 1255, 1511, 1605, 1701, 1733, 2972 cm^−1^; ^1^H-NMR (500.15 MHz, CDCl_3_): 1.89 (12H, d, J 7.3 Hz, C*H*3CH), 4.7 (4H, d J 7.0 Hz, inner OC*H*2O), 5.09 (8H, s, ArC*H*2), 5.16 (4H, q, J 7.1 Hz, CH_3_C*H*), 5.85 (4H, d J 7.1 Hz, outer OC*H*2O), 7.06 (8H, d, J 8.1 Hz, Ar), 7.46–7.81 (28H, m, Ar), 8.23 (8H, d, J 8.7 Hz, Ar). ^1^3C-NMR (125.78 MHz, CDCl_3_): 16.2 (*C*H_3_CH), 31.3 (CH_3_*C*H), 60.9 (O*C*H_2_O, 100.1 (Ar*C*H_2_O), 114.5, 118.7, 120.9, 121.2, 121.3, 122.6, 125.5, 126.4, 127.9, 129.31, 131.4, 132.5, 133.8, 139.1, 148.6, 162.8, 164.8 (Ar*C*=O).
**6**: Beige powder (70 mg, 50%), mp 235 °C. IR [cm^−1^] $\nu_{max}$(KBr): 976, 1008, 1167, 1260, 1509, 1605, 1736, 2933 cm^−1^; ^1^H-NMR (500.15 MHz, CDCl_3_): 0.94 (12H, s, ArC*H*3), 1.86 (12H, d, J 6.7 Hz, C*H*3CH), 1.17–3.19 (48H, m, steroid skeleton protons), 4.65 (4H, d J 5.8 Hz, inner OC*H*2O), 5.1 (8H, s, ArC*H*2), 5.13 (4H, q, J 6.6 Hz, CH_3_C*H*), 5.81 (4H, d J 6.6 Hz, outer OC*H*2O), 6.92–7.35 (16H, m, Ar), 7.45 (4H, s Ar), 8.16 (8H, d, J 6.5 Hz, Ar). ^1^3C-NMR (125.78 MHz, CDCl_3_): 8.5, 13.8, 16.2 (*C*H_3_CH), 21.6, 25.8, 26.39, 29.4, 32.3, 31.6 (CH_3_*C*H), 35.8, 38.0, 44.2, 47.9, 50.5, 60.8 (O*C*H_2_O, 100.0 (Ar*C*H_2_O), 112.9, 114.3, 115.3, 118.9, 121.8, 128.3, 132.4, 137.9, 139.1, 148.9, 154.0, 432.8, 164.9 (Ar*C*=O), 220.8 (*C*=O).
**7**: Beige powder (81.5 mg, 76%), mp 226 °C. IR [cm^−1^] $\nu_{max}$(KBr): 973, 1068, 1168, 1161, 1267, 1460, 1604, 1734, 2949 cm^−1^; ^1^H-NMR (500.15 MHz, CDCl_3_): 1.87 (12H, d, J 7.1 Hz, C*H*3CH), 3.94 (12H, s, C*H*3COO), 4.69 (4H, d, J 6.8 Hz, inner OC*H*2O), 5.05 (8H, s, ArC*H*2O), 5.13 (4H, q, J 7.6 Hz, CH_3_C*H*), 5.81 (4H, d, J 7.6 Hz, outer OCH2O), 7.04 (8H, d, J 8.8 Hz, Ar), 7.25 (8H, d, J 9.6 Hz, Ar), 7.47 (4H, s, Ar), 8.07 (8H, d, J 8.1 Hz, Ar), 8.17 (8H, d, J 8.17 Hz, Ar). ^1^3C-NMR (125.78 MHz, CDCl_3_): 16.2 (*C*H_3_CH), 31.3 (CH_3_*C*H), 52.2 (O*C*H_3_), 60.9 (O*C*H_2_O), 100.4 (Ar*C*H_2_O), 114.3, 121.03, 122.5 (overlapping signals), 122.6, 127.6, 131.1, 132.5, 139.1, 154.0, 154.9, 162.9, 164.0 (Ar*C*=O), 164.3 (O=*C*CH_3_).
**8**: Beige powder (55 mg, 49%), mp 235 °C. IR [cm^−1^] $\nu_{max}$(KBr): 978, 1008, 1094, 1255, 1509, 1736, 2971 cm^−1^; ^1^H-NMR (500.15 MHz, CDCl_3_): 1.89 (12H, d, J 7.5 Hz, C*H*3CH), 4.7 (4H, d, J 6.6 Hz, inner OC*H*2O), 5.04 (8H, s, ArC*H*2O), 5.15 (4H, q, J 6.6 Hz, CH_3_C*H*), 5.88 (4H, d, J 6.6 Hz, outer OCH2O), 7.12 (8H, d, J 9.8 Hz), 7.25–7.5 (28H, m, Ar), 7.72 (4H, d, J 8.9 Hz), 7.85 (8H, d, J 8.9 Hz), 8.31 (8H, d, J 8.9 Hz). ^1^3C-NMR (125.78 MHz, CDCl_3_): 16.2 (*C*H_3_CH), 31.3 (CH_3_*C*H), 60.9 (O*C*H_2_O), 100.0 (Ar*C*H_2_O), 108.6, 114.1, 114.5, 118.2, 118.3, 121.0, 121.2, 122.0, 122.4, 125.4, 125.9, 126.4, 127.0, 132.6, 134.6, 139.2, 146.8, 154.0, 164.7 (Ar*C*=O).
**9**: Light brown powder (75 mg, 70%), mp > 260 °C. IR [cm^−1^] $\nu_{max}$(KBr): 974, 1007, 1165, 1251, 1272, 1300, 1478, 1604, 1701, 1740, 2971 cm^−1^; ^1^H-NMR (500.15 MHz, CDCl_3_): 1.87 (12H, d, J 7.7 Hz, C*H*3CH), 3.8 (12H, s, ArOC*H*3), 4.69 (4H, d, J 7.5 Hz, inner OC*H*2O), 5.05 (8H, s, ArC*H*2O), 5.14 (4H, q, J 7.3 Hz, CH_3_C*H*), 5.84 (4H, d, J 6.9 Hz, outer OCH2O), 7.02 (8H, d, Ar), 7.07 (8H, d, J 8.5 Hz), 7.22–7.47 (16H, m, Ar), 8.21 (8H, d, J 8.3 Hz, Ar), 10.15 (4H, s, ArC*H*O). ^1^3C-NMR (125.78 MHz, CDCl_3_): 16.2 (*C*H_3_CH), 31.3 (CH_3_*C*H), 52.2 (O*C*H_3_), 60.9 (O*C*H_2_O), 100.4 (Ar*C*H_2_O), 114.1, 120.1, 121.0, 127.7, 131.2, 132.6, 138.3, 139.1, 154.0, 155.0, 162.9, 164.7 (Ar*C*=O), 166.3 (Ar*C*HO).
**10**: Beige powder (54 mg, 57%), mp > 260 °C. IR [cm^−1^] $\nu_{max}$(KBr): 976, 1008, 1166, 1200, 1250, 1264, 1300, 1511, 1605, 1732, 2972 cm^−1^; ^1^H-NMR (500.15 MHz, CDCl_3_): 1.87 (12H, d, J 7.3 Hz, C*H*3CH), 2.37 (12H, s, ArC*H*3), 4.7 (4H, d, J 6.6 Hz, inner OC*H*2O), 5.04 (8H, s, ArC*H*2O), 5.13 (4H, q, J 7.1 Hz, CH_3_C*H*), 5.81 (4H, d, J 7.2 Hz, outer OCH2O), 7.01–7.04 (12H, m, Ar), 7.15 (8H, d, J 8.4 Hz, Ar), 7.46 (4H, s, Ar), 8.15 (8H, d, J 8.4 Hz, Ar). ^1^3C-NMR (125.78 MHz, CDCl_3_): 16.2 (*C*H_3_CH), 20.9, 31.3 (CH_3_*C*H), 60.8 (O*C*H_2_O), 100.0 (Ar*C*H_2_O), 114.2, 120.8, 121.4, 122.6, 129.3, 132.4, 135.0, 139.1, 148.7, 154.0, 162.7, 164.8 (Ar*C*=O).
**11**: Brown powder (102 mg), mp > 260 °C. IR [cm^−1^] $\nu_{max}$(KBr): 978, 1008, 1064, 1266, 1475, 1606, 1744, 29720 cm^−1^; ^1^H-NMR (500.15 MHz, CDCl_3_): 1.85 (12H, br s, C*H*3CH), 2.09 (12H, s CH_3_), 2.10 (12H, s, CH_3_), 4.64 (4H, br s, inner OC*H*2O), 5.01 (8H, s, ArC*H*2O), 5.1 (4H, br s, CH_3_C*H*), 5.8 (4H, br s, outer OCH2O), 6.81–7.03 (20H, m, Ar), 7.45 (4H br s, Ar, 8.04 (4H, br s, Ar), 8.19 (8H, br s, Ar). ^1^3C-NMR (125.78 MHz, CDCl_3_): 16.2 (*C*H_3_CH), 31.3 (CH_3_*C*H), 46.2, 60.9 (O*C*H_2_O), 100.3 (Ar*C*H_2_O), 114.8, 120.9, 121.7, 125.7, 128.3, 129.4, 132.4, 139.1, 151.0, 154.1, 162,8, 164.6 (Ar*C*=O).
**12**: Grey powder (60-80 mg, isolated from product mixture). IR [cm^−1^] $\nu_{max}$(KBr): 973, 1090, 1160, 1245, 1482, 1597, 1685, 2134, 2876, 2968 cm^−1^; ^1^H-NMR (500.15 MHz, DMSO−d_6_): 1.89 (12H, d, J 6.9 Hz, $CH_{3}$CH), 4.44 (4H, q, J 7.6 Hz, CH_3_CH), 4.8–4.94 (12H, m, O$CH_{2}$O + Ar$CH_{2}$O), 5.78 (4H, d, J 7.0 Hz, outer O$CH_{2}$O), 7.05 (8H, d, J 8.8 Hz, Ar), 7.9 (12H, m, Ar). ^1^3C-NMR (125.78 MHz, DMSO−d_6_): 16.1 (*C*H_3_CH), 31.2 (CH_3_*C*H), 60.7 (Ar$CH_{2}$O), 100.0 (O*C*H_2_O), 114.3, 116.9, 120.7, 131.9, 138.4, 154, 158.4, 163.5, 171.5 (C=O). MS: 1293.3 [M]^+^.
**13**: Light grey powder (59 mg, 75%), mp 240–250 °C. IR [cm^−1^] $\nu_{max}$(KBr): 970, 1248, 1603, 1657, 2879, 2970 cm^−1^; ^1^H-NMR (500.15 MHz, DMSO−d_6_): 1.90 (12H, d, J 6.6 Hz, $CH_{3}$CH), 4.47 (4H, d, J 7.1 Hz, inner O$CH_{2}$O), 4.88 (2H, m, CH_3_CH + Ar$CH_{2}$O), 5.8 (4H, d, J 7.6 Hz, outer O$CH_{2}$O), 6.96 (8H, d, J 8.3 Hz, Ph), 7.1 (4H, br s), 7.8 (8H, d, J 8.3 Hz, Ph), 7.82 (4H, br s), 7.9 (4H, s). ^1^3C-NMR (125.78 MHz, DMSO−d_6_): 16.5 (*C*H_3_CH), 31.8 (CH_3_*C*H), 60.9 (Ar$CH_{2}$O), 99.8 (O*C*H_2_O), 114.4, 122.9, 127.1, 129.9 (overlapping signals), 139.5, 153.6, 161.2, 168.1 (C=O). MS: 1189.3 [M]^+^.

## Figures and Tables

**Table 1:** Effect of temperature on conversion. The reactions were carried out at atmospheric pressure; the reaction time was 24 h.

Run	Temperature (℃)	Phosphine	Conversion (%)
1	50	PPh_3_	<5
2	75	PPh_3_	32
3	100	PPh_3_	100
4	50	Xantphos	<5
5	75	Xantphos	38
6	100	Xantphos	100

**Table 2:** The achieved chemoselectivities for different ligands and nucleophiles at atmospheric pressure and 100 °C.

Run	Cavitand	Phosphine	Chemoselectivity (%)
1	**2**	PPh_3_	72
2	**2**	Xantphos	85
3	**3**	PPh_3_	65
4	**3**	Xantphos	77
5	**4**	PPh_3_	83
6	**4**	Xantphos	77
7	**5**	PPh_3_	68
8	**5**	Xantphos	78
9	**6**	PPh_3_	75
10	**6**	Xantphos	72
11	**7**	PPh_3_	82
12	**7**	Xantphos	100
13	**8**	PPh_3_	58
14	**8**	Xantphos	74
15	**9**	PPh_3_	70
16	**9**	Xantphos	90
17	**10**	PPh_3_	90
18	**10**	Xantphos	75

**Table 3:** Chemoselectivities and conversions of azidocarbonylation at atmospheric pressure.

Run	Temp. (℃)	Cat. Ratio (mol%)	Reac. Time	Conv. (%)	Chemosel. (%)	Product
1	50	2	16 h	100	100	**13**
2	rt	4	16 h	51	100	**12**
3	rt	8	16 h	100	66	**12** + **13**
4	rt	2	2 days	32	100	**12**
5	rt	8	2 days	100	63	**12** + **13**
6	rt	8	3 days	100	59	**12** + **13**

## References

[1] Cram D.J., Cram J.M. (1974). Host-Guest Chemistry: Complexes between organic compounds simulate the substrate selectivity of enzymes. Science.

[2] Moran J.R., Karbach S., Cram D.J. (1982). Cavitands: Synthetic molecular vessels. J. Am. Chem. Soc..

[3] Cram D.J. (1992). Molecular container compounds. Nature.

[4] Cram D.J., Cram J.M. (1997). Container Molecules and Their Guests.

[5] Timmerman P., Verboom W., Reinhoudt D.N. (1996). Resorcinarenes. Tetrahedron.

[6] Ma S., Rudkevich D.M., Rebek J. (1998). “Deep-Cavity” resorcinarenes dimerize through hydrogen bonding and self-inclusion. J. Am. Chem. Soc..

[7] Aakeröy C.B., Schultheiss N., Desper J. (2006). C-Pentyltetra (3-pyridyl) cavitand: A versatile building block for the directed assembly of hydrogen-bonded heterodimeric capsules. Org. Lett..

[8] Hass O., Schierholt A., Jordan M., Lützen A. (2006). Improved synthesis of monohalogenated cavitands and their use in the synthesis of further functionalized cavitands. Synthesis.

[9] Lin Z., Emge T.J., Warmuth R. (2011). Multicomponent Assembly of Cavitand-Based Polyacylhydrazone Nanocapsules. Chem.-Eur. J..

[10] Yu J.T., Huang Z.T., Zheng Q.Y. (2012). Synthesis, structure, fullerene-binding and resolution of C 3-symmetric cavitands with rigid and deep cavities. Org. Biomol. Chem..

[11] Ogoshi T. (2012). Synthesis of novel pillar-shaped cavitands “Pillar [5] arenes” and their application for supramolecular materials. J. Incl. Phenom. Macrocycl. Chem..

[12] Sémeril D., Matt D., Ramesh R. (2019). Synthesis of the first resorcin [4] arene-functionalized triazolium salts and their use in Suzuki–Miyaura cross-coupling reactions. Catalysts.

[13] Kégl T.R., Kégl T. (2020). Palladium-catalyzed carbonylative synthesis and theoretical study of elongated tubular cavitands. J. Organomet. Chem..

[14] Jánosi T.Z., Makkai G., Kégl T., Mátyus P., Kollár L., Erostyák J. (2016). Light-Enhanced Fluorescence of Multi-Level Cavitands Possessing Pyridazine Upper rim. J. Fluoresc..

[15] Kégl T., Csekő G., Mikle G., Takátsy A., Kollár L., Kégl T. (2017). The Role of Weak Interactions in Supramolecular Compounds: A Synthetic and Theoretical Study of Novel Elongated Cavitands. ChemistrySelect.

[16] Csók Z., Takátsy A., Kollár L. (2012). Highly selective palladium-catalyzed aminocarbonylation and cross-coupling reactions on a cavitand scaffold. Tetrahedron.

[17] Wan Y., Song F., Ye T., Li G., Liu D., Lei Y. (2019). Carbonylative Suzuki coupling and alkoxycarbonylation of aryl halides using palladium supported on phosphorus-doped porous organic polymer as an active and robust catalyst. Appl. Organomet. Chem..

[18] Geng H.Q., Wu X.F. (2021). Copper-Catalyzed Alkoxycarbonylation of Alkyl Iodides for the Synthesis of Aliphatic Esters: Hydrogen Makes the Difference. Org. Lett..

[19] Mikle G., Noveczky P., Mahó S., Kollár L. (2021). Palladium-catalysed amino-vs. Alkoxycarbonylation of iodoalkenes using bifunctional N, O-nucleophiles. Tetrahedron.

[20] Liu N., Wu X., Wang C., Qu J., Chen Y. (2022). Nickel-catalyzed alkoxycarbonylation of aryl iodides with 1 atm CO. Chem. Commun..

[21] Kégl T.R., Mika L.T., Kégl T. (2022). 27 Years of Catalytic Carbonylative Coupling Reactions in Hungary (1994–2021). Molecules.

[22] Brennführer A., Neumann H., Beller M. (2009). Palladium-catalyzed carbonylation reactions of aryl halides and related compounds. Angew. Chem. Int. Ed. Eng..

[23] Wu X.F., Neumann H., Beller M. (2011). Palladium-catalyzed carbonylative coupling reactions between Ar–X and carbon nucleophiles. Chem. Soc. Rev..

[24] Satoh T., Ikeda M., Miura M., Nomura M. (1996). Palladium-catalyzed phenoxycarbonylation of aryl iodides: Electronic effect of the substituents on phenol. J. Mol. Catal. A-Chem..

[25] Watson D.A., Fan X., Buchwald S.L. (2008). Carbonylation of aryl chlorides with oxygen nucleophiles at atmospheric pressure. Preparation of phenyl esters as acyl transfer agents and the direct preparation of alkyl esters and carboxylic acids. J. Org. Chem..

[26] Tukacs J.M., Marton B., Albert E., Tóth I., Mika L.T. (2020). Palladium-catalyzed aryloxy-and alkoxycarbonylation of aromatic iodides in *γ*-valerolactone as bio-based solvent. J. Organomet. Chem..

[27] Szuroczki P., Molnár L., Dörnyei Á., Kollár L. (2020). Facile, High-Yielding Synthesis of 4-Functionalised 1, 2, 3-Triazoles via Amino-and Aryloxycarbonylation. ChemistrySelect.

[28] Seni A.A., Kollar L., Mika L.T., Pongracz P. (2018). Rhodium-catalysed aryloxycarbonylation of iodo-aromatics by 4-substituted phenols with carbon monoxide or paraformaldehyde. Mol. Catal..

[29] Miloserdov F.M., Grushin V.V. (2012). Palladium-Catalyzed Aromatic Azidocarbonylation. Angew. Chem. Int. Ed. Eng..

[30] Miloserdov F.M., McMullin C.L., Belmonte M.M., Benet-Buchholz J., Bakhmutov V.I., Macgregor S.A., Grushin V.V. (2014). The challenge of palladium-catalyzed aromatic azidocarbonylation: From mechanistic and catalyst deactivation studies to a highly efficient process. Organometallics.

[31] Yadav V.K., Srivastava V.P., Yadav L.D.S. (2016). One-Pot Synthesis of Carbamoyl Azides via Palladium-Catalysed Azidocarbonylation of Haloarenes Using N-Formylsaccharin as a CO Surrogate. Synlett.

[32] Li M., Yu F., Chen P., Liu G. (2017). Palladium-catalyzed intermolecular azidocarbonylation of alkenes via a cooperative strategy. J. Org. Chem..

[33] Mikle G., Skoda-Földes R., Kollar L. (2021). Amino-and azidocarbonylation of iodoalkenes. Tetrahedron.

[34] Sharma A.K., Bhattacherjee D., Sharma N., Giri K., Das P. (2021). Supported-Pd catalyzed tandem approach for N-arylbenzamides synthesis. Mol. Catal..

[35] Csók Z., Kégl T., Li Y., Skoda-Földes R., Kiss L., Kunsági-Máté S., Todd M.H., Kollár L. (2013). Synthesis of elongated cavitands via click reactions and their use as chemosensors. Tetrahedron.

[36] Tunstad L.M., Tucker J.A., Dalcanale E., Weiser J., Bryant J.A., Sherman J.C., Helgeson R.C., Knobler C.B., Cram D.J. (1989). Host-guest complexation. 48. Octol building blocks for cavitands and carcerands. J. Org. Chem..

[37] Román E., Peinador C., Mendoza S., Kaifer A.E. (1999). Improved Synthesis of Cavitands. J. Org. Chem..

[38] Sorrell T.N., Pigge F.C. (1993). A convenient synthesis of functionalized cavitands via free-radical bromination. J. Org. Chem..

[39] Csók Z., Kégl T., Párkányi L., Varga Á., Kunsági-Máté S., Kollár L. (2011). Facile, high-yielding synthesis of deepened cavitands: A synthetic and theoretical study. Supramol. Chem..

[40] Körner S.K., Tucci F.C., Rudkevich D.M., Heinz T., Rebek Jr J. (2000). A Self-Assembled Cylindrical Capsule: New Supramolecular Phenomena through Encapsulation. Chem. Eur. J..

[41] Gangemi C.M.A., Pappalardo A., Trusso Sfrazzetto G. (2015). Assembling of supramolecular capsules with resorcin [4] arene and calix [n] arene building blocks. Curr. Org. Chem..

[42] Galan A., Ballester P. (2016). Stabilization of reactive species by supramolecular encapsulation. Chem. Soc. Rev..

